# Perceived risk versus objectively measured risk of HIV acquisition: a cross-sectional study among HIV-negative individuals in Serodiscordant partnerships with clients attending an Urban Clinic in Uganda

**DOI:** 10.1186/s12889-019-7929-0

**Published:** 2019-11-29

**Authors:** Lillian Tugume, Timothy Ronald Muwonge, Edith Nakku Joloba, John Bosco Isunju, Flavia Matovu Kiweewa

**Affiliations:** 1 Makerere University College of Health Sciences, School of Public Health, Kampala, Uganda; 2 Infectious Diseases Institute, Makerere University, Kampala, Uganda; 3 Makerere University- John Hopkins University Research Collaboration, Kampala, Uganda

**Keywords:** HIV, Pre-exposure prophylaxis, Risk perception

## Abstract

**Background:**

Acceptability of Pre-exposure Prophylaxis (PrEP) could be hampered by low self-perceived risk for HIV acquisition. Moreover, discordance between risk perception and actual risk of HIV acquisition is likely to occur. We assessed congruence between the level of self- perceived and that of objectively scored risk of HIV acquisition among HIV-negative individuals in discordant relationships.

**Methods:**

This was a cross-sectional study among a representative sample of HIV-negative adult males and females whose partners were receiving antiretroviral therapy for at least 3 months from the Infectious Diseases Institute Clinic in Kampala, Uganda. Perceived risk was measured based on self-report using a numerical rating scale whereas objective risk was measured using a validated risk score tool. Congruence between perceived risk and objectively scored risk was evaluated using descriptive statistics and validity measures. Incongruence between the two phenomena was further evaluated using univariate and multivariate regression analyses.

**Results:**

HIV-negative partners evaluated in this study were mostly male (64%) with a median age of 41 years (IQR 35 to 50). Majority (76.3%) of the partners perceived themselves as low risk for HIV acquisition. Similarly, most (93.8%) were objectively scored as low risk. However, nearly three quarters (72.7%) of partners who were objectively scored as high risk perceived themselves as being at low risk and all were men. The sensitivity and specificity of perceived risk for detecting the objectively measured risk was 27.3 and 76.5% respectively; area under ROC curve = 0.52; 95%CI (0.38, 0.66). The proportion of participants at high risk of HIV acquisition who perceived their risk as low was greater among those whose partners had detectable viral load compared to participants whose partners had undetectable viral load (PR = 0.51; 95%CI 0.29 to 0.90).

**Conclusion:**

Incongruence between perceived and objectively measured risk of HIV acquisition does occur especially among individuals whose partners had a detectable viral load. PrEP counselling for serodiscordant couples should focus on explaining the consequence of detectable viral load in the HIV-positive partner on HIV transmission risk.

## Background

The focus of pre-exposure prophylaxis (PrEP) research after establishing efficacy for averting new Human Immunodeficiency Virus (HIV) infections has been on demonstration and implementation projects in the last 5 years [1, 2]. However, the success of large-scale PrEP implementation requires ongoing consideration of broader contextual issues. Self-perception of HIV acquisition risk by potential PrEP users is one such issue thought to drive PrEP uptake [3]. This is mainly because motivation for adherence to HIV prevention methods is largely determined by one’s perception of likelihood of HIV acquisition [4, 5]. Paradoxically, participants in a PrEP demonstration study conducted in Uganda and Kenya reported generally low perceived risk of HIV acquisition but had high levels of initiating and adhering to PrEP [6]. We speculate that the fact that index partners had recently initiated antiretroviral therapy (ART), could have been a motivating factor resulting in high acceptability. Moreover, risk perception is likely to be determined by multifaceted social and psychological factors [6]. One could argue that the perceived risk of HIV transmission and acquisition could be altered by the fact that HIV-positive individuals are initiated on ART as soon as they are tested in keeping with the “test and start” strategy. In fact, HIV-negative women in serodiscordant relationships who had early declining adherence in one study took less PrEP doses in the weeks when the partner was on ART compared to the weeks when the partner was not on ART [7]. It is possible that they may have taken less PrEP doses in the period when they perceived themselves to be at lower risk of HIV acquisition i.e. when the partner was receiving ART.

Objective risk factors of HIV transmission among serodiscordant couples include high HIV viral load in the HIV-positive partner, unprotected sexual activity, multiple partners and circumcision status for HIV-negative male partners [8–10]. While HIV transmission risk is expected to be highest during the first 6 months of initiating ART, substantial risk may still exist even when the HIV-positive partner has been on ART for over 6 months. Notably, up to 7.4% of 559 individuals on ART for over 6 months in a Ugandan cohort never attained viral suppression and 31.7% of those who did, experienced virologic failure [11]. In the setting of ART for the HIV-positive partner, transmission risk, though lower, still persists in the first 6 months of ART initiation due to incomplete viral suppression [12]. While all HIV-negative partners in serodiscordant relationships are at high risk for HIV acquisition, a validated risk score has been developed primarily for research purposes to identify highest risk couples in order to maximize efficiency and cost-effectiveness of PrEP [13, 14]. From this score, internal and external validation showed similar predictive ability of the risk score even when viral load was excluded from the risk score [13, 14].

However, it is possible that high risk individuals may underestimate their risk as not being significant enough to warrant PrEP use [15, 16]. Furthermore, discordance between perceived risk and objective risk of HIV acquisition does occur and has been studied largely among men who have sex with men [16, 17]. Whether a similar observation is expected in serodiscordant couples and the plausible explanation for such an observation is less clear. A sub study of the partners PrEP study revealed that reporting unprotected sex was a predictor of high perceived risk [6], however predictors of incongruence between perceived and objective risk are unclear. Also, the study recruited participants who were already part of an HIV prevention trial and had received PrEP for at least 6 months; hence the need to evaluate these phenomena in serodiscordant couples who have apparently varied risk of HIV transmission. In this study, we evaluated congruence between self-perceived and objectively measured HIV transmission risk among partners in serodiscordant relationships including those that are at apparently lower risk of HIV acquisition i.e. where the HIV-positive partner is already receiving antiretroviral therapy. Secondly, we sought to determine the predictors of congruence between perceived and measured risk of HIV acquisition among HIV-negative individuals in serodiscordant relationships with individuals who are receiving ART.

## Methods

This cross-sectional study was conducted between March 2018 and July 2018 at the Infectious Diseases Institute (IDI) Mulago; an urban clinic and center of excellence for prevention, care and treatment of HIV in Uganda. Notably, this study was conducted prior to roll out of pre-exposure prophylaxis (PrEP) in the clinic. A random sample of heterosexual couples in a serodiscordant partnership was selected from the cohort of stable (i.e. those who expected to remain together ≥24 months and those with some sexual activity in the last 3 months) serodiscordant couples in the clinic to participate in the study. The underlying dynamic cohort from which couples were sampled is captured in the clinic’s database. Couples are typically followed up every quarter for HIV testing and counselling on HIV prevention. HIV-negative partners who were 18 years or older and whose partners were receiving antiretroviral therapy for 3 months or longer were included in the study. However, individuals to whom HIV status of partner had not been disclosed and couples with no intentions to engage in a sexual relationship were excluded.

Regarding sampling, a list of all serodiscordant couples (*N* = 737) was obtained from the clinic’s data base, followed by stratifying couples by viral load status of the HIV-positive partner i.e. detectable versus undetected. Participants for each stratum were then selected by proportionate stratified sampling. The study was powered around the primary question which is unpublished. The primary question was to determine the proportion of HIV-negative partners that were willing to take up PrEP. We assumed that the expected proportion would be 81% [18], a normal standard variate of 1.96 at 5% type 1 error, and precision of 5%. Using the Kish Leslie sample size formula for cross-sectional studies where the outcome is a proportion, we obtained a sample size of 236 HIV negative partners [19]. On adjusting for a finite population of 737 couples, the minimum sample size would be 160. A total of 180 participants were targeted after taking into account a non-response rate of 10%. Of the 737 HIV-negative partners,13.9% (102/737) had partners with detectable viral load while 86.1% (635/737) had partners with undetected viral load. We stratified by viral-load status of the HIV positive partner as this was a measurable marker of HIV transmission risk that was available at the outset. Our goal was to have a sample of HIV-negative individuals with varied HIV acquisition risk as opposed to having one with mostly low risk. Of the 180 sampled, 25 had partners with detectable viral load while 155 had partners with undetected viral load.

A structured questionnaire was administered to eligible partners by a nurse or counselor and these health care workers were working in the clinic. Prior to administering the questionnaire, we explained to participants what PrEP is and how effective it is using layman’s terms. We defined “perceived risk of HIV acquisition” as a lay person understanding of one’s likelihood to acquire HIV from the HIV-positive partner. Conversely, objectively measured risk was defined as the likelihood of HIV transmission from the HIV-positive partner to the negative partner as assessed by a validated risk scoring tool. Perceived risk of HIV acquisition was measured using an eleven-point numerical rating scale (NRS-11), adopted from pain scale whereas objective risk was scored using a validated risk score tool [13, 14]. Answer choices 0 to 3 to the NRS-11 scale were categorized as low risk perception whereas choices 4 to 10 were categorized as high risk. Regarding measurement of “objective risk, “the tool comprises of six easily measurable items. These include; age, number of children, marital status, male circumcision status, condom use and plasma HIV-1 levels of the HIV-positive partner.

Furthermore, we conducted chart reviews to obtain the most recent viral load, ART regimen and age of the HIV-positive partner. Individuals with viral load greater than 75 copies/ml were classified as having detectable viral load as this is the lower limit of detection for the assay. For persons who had been on ART for less than 6 months, we assumed that their viral load was detectable since viral loads are not routinely obtained unless one has been on ART for over 6 months [20–22].

Ethical approval for this study was granted by the “Higher Degrees Research and Ethics Committee” of Makerere University School of Public Health and administrative clearance granted by the IDI scientific review committee. All participants provided verbal consent prior to participation in the study.

### Statistical analysis

Descriptive statistics were used to assess the level of congruence between perceived risk and objectively scored risk. Participants were categorized into four groups; two congruent and two incongruent groups in relation to measured and perceived risk. Participants whose perceived risk was the same as measured risk were categorized as congruent whereas participants whose perceived risk differs from measured risk were categorized as incongruent. Proportions of participants in the four groups were obtained as follows; 1) Individuals who perceive themselves as low risk that are categorized as low risk using the validated risk score, 2) Individuals who perceive themselves as high risk that are categorized as high risk using the validated risk, 3) Individuals who perceive themselves as low risk that are categorized as high risk using the validated risk score and 4) Individuals who perceive themselves as high risk that are categorized as low risk using the validated risk score. We further utilized measures of accuracy including sensitivity, specificity and area under receiver operating characteristic curve to evaluate risk perception (perceived risk) against measured risk (objective risk score tool) as the reference standard. Finally, covariates of congruence between perceived risk and objectively scored risk of HIV acquisition were assessed in bivariate and multivariate modified Poisson regression analyses. We used stepwise regression method for model selection. Covariates that were significant at *P* < 0.2 were included in the multivariate analysis. All other analyses were evaluated against a two-sided alpha level of 0.05. Statistical analyses were conducted in Stata software (StataCorp. 2013. Stata Statistical Software: Release 13. College Station, TX: StataCorp LP).

## Results

### Study population

The total number of partners who were sampled was 180, however, two were found to have separated and one was living abroad at the time of study enrollment giving a total analytical sample of 177 individuals. All the three partners who did not respond had partners with detectable viral load. Majority (64%) of the respondents were women and the overall median age was 41 years; IQR (35–50), Table 1. At the time of the survey, the HIV-positive partners had been on ART for a median duration of 7.7 years; IQR (4.8–11.1) and majority (88%) were on a regimen considered as first line in the clinic. Less than half (41%) of the partners knew whether their HIV-positive partner had detectable or undetectable viral load.

**Table 1:** Socio-demographic and behavioral characteristics of the study population and summary of partners’ ART history

	Summary statistic *N* = 177
Variable	n (%) or median (IQR)
Age in years	41 (35,50)
Age group	
< =45 years	118 (66.7)
> 45 years	58 (33.5)
Men	63 (36)
Monogamous relationship	156 (88)
No. of Children	4 (2,6)
Desire to conceive	90 (51)
Reporting condom-less sex	61 (35)
Aware of partner’s Viral load status	72 (41)
Circumcised men	35 (60)
Partner’s ART history	
First line Regimen	155 (88)
Duration on ART in years	7.7 (4.8,11.1)
^a^Detectable viral load	22 (13)
Time since last viral load in months	6 (3,9)

*Abbreviations*: *ART* Anti-retroviral therapy, ^a^Detectable viral load is defined as Viral Load > 75copies/ml

### Accuracy of perceived risk as compared to objectively scored risk of HIV acquisition

Overall, 6.2% (11/177) of the participants were categorized as being at high risk of HIV infection using the objective risk score tool whereas 23.7% (42/177) of the participants in this survey perceived themselves as having a high risk of acquiring HIV from their partners, Table 2**.** Among those who had high perceived risk, only 7.1% (*N* = 42) were objectively scored as high risk and among those who had low perceived risk, 94.1% (*N* = 135) were objectively scored as low risk. Of note, 72.7% (*N* = 11) of those who were objectively scored as high risk perceived themselves as low risk.

**Table 2:** Comparison of objectively scored risk score and perceived risk for categorizing risk of HIV acquisition

		Objective Risk Score ^a^		
		High	Low	
Perceived risk	High	3	39	PPV 7.14%
	Low	8	127	NPV 94.10%
		Sensitivity 27.27%	Specificity 76.51%	

^a^Objective risk score is assumed to be the reference standard. *Abbreviations*: *PPV* Positive Predictive Value, *NPV* Negative predictive value. Area under ROC curve = 0.52; 95%CI (0.38, 0.66)

### Predictors of congruence between perceived risk and objectively scored risk of HIV acquisition

Objectively scored risk and risk perception were congruent in 73.4%(130/177) of the participants and incongruent in 26.6% (47/177) of the participants. Participants who were categorized as low risk by both methods were mostly women (86/127) aged 45 years or younger (81/126). Most of their partners (119/127) had undetectable viral load and had been on ART for 10 years or less (79/127). On the contrary, participants who perceived themselves as low risk but were objectively scored as high were all men (8/8). Majority of their partners had detectable viral load (6/8) and had been on ART for less than 10 years (6/8). Only 25% (31/124) of participants categorized as low risk by both methods reported condom-less sex whereas most (6/8) of the participants objectively scored as high but with low perceived risk reported engaging in condom-less sex with their partners, Table 3**.**

**Table 3:** Differences in descriptive characteristics by objective risk category among individuals with low risk perception

Variable	Low perceived &low objective (*N* = 127)	Low perceived and high objective (*N* = 8)	Fisher’s exact *P*-value
Men	41	8	< 0.001
Age < =45	81	7	0.263
circumcised	61	3	0.718
^a^Detectable viral load (partner)	8	6	< 0.001
Partner’s ART duration			
< =10 years	48	2	0.710
Reporting condom-less sex	31	6	0.006

^a^Detectable viral load is defined as Viral Load > 75copies/ml

The proportion of participants who were rated similarly by both methods was less by half among those whose risk was objectively scored as high relative to those whose risk was objectively scored as low (PR = 0.52; 95%CI: 0.31–0.86). Additionally, participants whose partners had detectable viral load were less likely to have congruence between measured and perceived risk. Neither being aware of partner’s viral load status nor education level was predictive of congruence.

In the multivariate analysis, congruence was significantly associated with viral load status of the HIV-positive partner after adjusting for high objectively measured risk, Table 4.

**Table 4:** Factors associated with congruence between perceived risk and measured risk of HIV acquisition

	Crude PR; 95% CI	^a^Adjusted PR; 95%CI
Age < =45	0.87 (0.73,1.03)	
Education level		
secondary	0.97 (0.81,1.17)	
tertiary	0.79 (0.58,1.09)	
none	0.85 (0.53,1.38)	
Polygamous relationship	1.18 (0.97,1.44)	
Aware of partner’s viral load status	1.12 (0.95,1.33)	
On ART > 10 years	0.90 (0.74,1.08)	
Partner has ^b^detectable viral load	0.64 (0.50,0.81) ^†††^	0.51 (0.29–0.90^) †^
High measured risk (objective)	0.48 (0.22,1.04)	0.65 (0.30–1.40)

^a^Adjusted estimates obtained from generalized linear model of binomial family

^b^Detectable viral load defined as viral load>75 copies/ml. †*P*-value < 0.05, †††*P*-value< 0.001

## Discussion

We observed that a low proportion of HIV-negative partners in this study were objectively scored to be at high risk of HIV transmission and would therefore be prioritized for pre-exposure prophylaxis (PrEP). The small percentage of serodiscordant couples considered to be at relatively higher risk of HIV transmission is not surprising as the Infectious Diseases Institute (IDI) is a center of excellence with a leak proof mechanism to ensure that at least 90% of clients on antiretroviral therapy (ART) are virally suppressed [22, 23]. In fact, in our study population, only 13% of the HIV-positive partners had a detectable viral load and this proportion would be lower if a more conservative cut-off for detectable viral load (> 1000 copies/ml) were considered. Additionally, the median time since the most recent viral load was 6 months implying that viral load monitoring is optimal at this clinic. This is in stark contrast to a landmark PrEP implementation study in Uganda and Kenya where 32% of the negative partners were scored as high risk [14, 24]. Enrollment was conducted at both rural and urban HIV clinics [18].

With regard to risk perception, nearly a quarter of the HIV-negative partners perceived themselves to be at high risk of HIV acquisition. Paradoxically, majority of the HIV-negative partners that perceived themselves as high risk were objectively scored as low risk. One plausible explanation would be that this subgroup of partners might consider themselves to be at high risk because of other sexual relations. Furthermore, objectively scored risk and perceived risk were congruent in most cases and incongruent in over one quarter of the cases, implying under/over estimation of one’s HIV acquisition risk. Likewise, it has been previously observed that women who are at substantial risk of acquiring HIV often underestimate their risk [25, 26]. The two methods are not expected to be 100% congruent as individual perspective of risk is often informed by psycho-social and socio-cultural dimensions [27]. On the other hand, public health approaches such as objective risk assessment are often based on scientific evidence and in the context of PrEP, the aim is to optimize cost-effectiveness [14]. The concept of risk perception is important because risk perception has been shown to be associated with adherence [28]. On the contrary, partners in the partners’ PrEP demonstration study had mostly low risk perception but adherence to PrEP was high [13, 18]. This is probably because the partners were assessed for risk perception 12 months after initiating PrEP and their perception at that point could have been different from their perception before initiating PrEP and shortly after.

If risk perception were truly related to adherence, the consequence of persons who are at high risk of HIV acquisition but perceive themselves as low is expected to be direr from a program perspective than that of persons who are at low risk and perceive themselves as high risk. In the population under study, we observed that nearly three quarters (72.7%) of partners who were objectively scored as high risk perceived themselves as low risk and all were men. It is possible that this same percentage would have poor retention and adherence if they were initiated on PrEP based on their high risk score with no intervention to address risk perception. Assuming that objectively scored risk of HIV transmission is the reference standard for categorizing HIV acquisition risk, our findings suggest that self-perception of risk has low sensitivity and positive predictive value for detecting the actual risk. Certainly we know that predictive value of a test decreases when it is used in a low prevalence population such as the population under study where only 6.2% were objectively scored as being at high risk [29]. Nonetheless, the sensitivity suggests that the probability of perceiving oneself as being at high risk when the partner is indeed at high risk of HIV acquisition is only 27.3%. While it might be true that people generally think they are okay when in reality they are not; this phenomenon may underlie poor adherence to daily PrEP in some key populations such as young women [30, 31].

In particular, the proportion of HIV-positive partners with detectable viral load was significantly higher among high risk HIV-negative partners that perceived themselves as low compared to low risk partners that rightly perceived themselves as low. Furthermore, detectable viral load in the HIV-positive partner remained a significant predictor of incongruence between objective and perceived risk after adjusting for awareness of partner’s viral load status. In view of the above, one plausible hypothesis is that partners attribute their risk of HIV acquisition to mostly unsafe sexual behaviour and not viral load status of their partners or a combination of the two. It is possible that partners even when aware of viral load suppression status may not fully comprehend the impact of viral load suppression or lack of it on the risk of HIV transmission.

The main limitation of our study is non-representativeness of a typical public health facility in Uganda as the study was conducted at a center of excellence for prevention, care and treatment of HIV. The proportion of HIV-negative partners who are considered to be at high risk of HIV acquisition is expected to be lower in a population where under 10% of the HIV-positive partners have a detectable viral load compared to a population with a detectable viral load prevalence > 10%. The latter scenario would be expected in a typical public health facility. However, the advantage is that this study gives perspective on risk assessment in the context of an effective HIV treatment program (> 90% viral load suppression rate in HIV-positive partners). Finally, data on willingness to use PrEP was assessed by self-report and there-fore potentially subject to social-desirability bias. This bias could potentially inflate the proportion of partners reporting safe sex. However, PrEP in Uganda is currently being offered primarily through health facilities and if social desirability bias does occur, it is likely to occur in a real world setting as well.

## Conclusions

Incongruence between perceived and objectively measured risk of HIV acquisition does occur especially among individuals whose partners had a detectable viral load. PrEP counselling for serodiscordant couples should focus on explaining the significance of detectable viral load in the HIV-positive partner as far as HIV transmission risk is concerned.

In summary, our findings provide formative insight regarding risk perception among a sub-group of serodiscordant couples that have previously not been the focus of implementation studies i.e. couples where the HIV-positive partner has been receiving ART long term.

## Data Availability

The datasets supporting the conclusions of this article are available from the corresponding author on reasonable request.

## Abbreviations

ART: Antiretroviral therapy
HIV: Human Immunodeficiency Virus
IDI: Infectious Diseases Institute
NRS-11: 11-point Numerical Rating Scale
PrEP: Pre-exposure prophylaxis
